# EHMTI-0086. Pure mestrual vestibular migraine

**DOI:** 10.1186/1129-2377-15-S1-D43

**Published:** 2014-09-18

**Authors:** C Mostardini, G Nola, R Giovanni

**Affiliations:** 1 Neurology Department, Giovan Battista Grassi Hospital, Roma, Italy; 2 Otolaringology Department, Giovan Battista Grassi Hospital, Roma, Italy; 3 Sapienza, University of Rome Dept of Otolaryngology, Italy

## 

We describe two cases of a young patients affected, by benign paroxysmal positional vertigo (BPPV) during menstruation.

Patient 1, of 32, present BPPV every month with menstruation, associated with constrictive non pulsating mild headache. Patient 2, of 43, present BPPV 6/7 time/year always during menstruation associated with severe throbbing headache.

Both patients present history of kinetosis, familiarity for migraine, absence of vertigo and headache out of menstruation or during pregnancy.

For a lot of years they were followed by otolaryngology for BPPV and treated with canalith repositioning procedure, without results. Instrumental examination like brain MRI and audiometric tests were normal, only nystagmus was present every crisis.

Occurrence of the vertigo/headache and neurovegetative symptoms only during menstruation, we conclude for the diagnosis of Vestibular Migraine (VM) and treated like a pure menstrual migraine. Patient 1 undergone to a short term prophylactic therapy with naproxene 550mg bid starting before the period until the fourth day and frovatriptan 2,5mg like rescue therapy, patients 2 for the unpredictability of the crisis should start with rizatriptan or naproxene followed by short term prophylactic like patient 1. Both patients respectively after 4 and 6 month of observation, are free from headache and vertigo.

This case represent the important overlap between migraine and vertigo defined VM by International Headache Society. This overlap and its treatment is usually well recognized when headache is the prevalent symptom but is important improve collaboration between headache expert and otolaryngology, to always recognize a VM and treat it in the correct way.

No conflict of interest.

